# 三肽聚合物基细胞印迹水凝胶对循环肿瘤细胞的高效捕获

**DOI:** 10.3724/SP.J.1123.2025.05002

**Published:** 2026-02-08

**Authors:** Wenjing SUN, Zhiyuan ZHANG, Xinmiao ZHAO, Jinghua CHEN, Guangyan QING

**Affiliations:** 1. 江南大学生命科学与健康工程学院，江苏 无锡 214122; 1. School of Life Science and Health Engineering，Jiangnan University，Wuxi 214122，China; 2. 中国科学院大连化学物理研究所，辽宁 大连 116023; 2. Dalian Institute of Chemical Physics，Chinese Academy of Sciences，Dalian 116023，China; 3. 江南大学化学与材料工程学院，江苏 无锡 214122; 3. School of Chemical and Material Engineering，Jiangnan University，Wuxi 214122，China; 4. 辽宁师范大学化学化工学院，辽宁 大连 116029; 4. School of Chemistry and Chemical Engineering，Liaoning Normal University，Dalian 116029，China

**Keywords:** 循环肿瘤细胞, 细胞印迹, 水凝胶, 界面, 富集, circulating tumor cells, cell imprinting, hydrogels, interfaces, enrichment

## Abstract

循环肿瘤细胞（circulating tumor cells， CTC）作为癌症转移的关键介质，在血液中的含量极低，其高效捕获对癌症早期诊断与治疗监测具有重要意义。然而，现有检测技术普遍存在抗体依赖性强、血液基质干扰严重等局限性，亟需开发具有高选择性和良好生物相容性的新型富集材料。本研究创新性地结合细胞印迹技术与氨基酸亲和协同策略，设计了一种基于色氨酸-组氨酸-精氨酸（tryptophan-histidine-arginine， Try-His-Arg， WHR）三肽聚合物的细胞印迹水凝胶材料，用于CTC的高效捕获。我们首先制备了多孔结构的介孔二氧化硅（mesoporous silica nanoparticles， MSN）作为载体，通过硅烷偶联剂修饰环氧基团后，与WHR三肽进行开环反应，成功合成了WHR@SiO₂功能材料。WHR@SiO₂对唾液酸（*N*-acetylneuraminic acid， Neu5Ac）及唾液酸化糖肽（sialylated glycopeptide， SGP）表现出显著亲和力。在此基础上，我们以聚乙二醇二甲基丙烯酸酯（poly（ethylene glycol） dimethacrylate， PEGDMA）为骨架，通过自由基聚合制备了具有模板细胞匹配三维微结构的WHR修饰细胞印迹水凝胶。实验结果表明，所制备的WHR水凝胶对SMMC-7721细胞的捕获效率高达94%，显著优于单一氨基酸修饰的水凝胶，同时展现出优异的血液相容性。材料对人血清白蛋白（human serum albumin， HSA）的吸附率低于5%，表明其具有出色的抗污能力。细胞毒性评估显示，WHR水凝胶具有良好的生物相容性，细胞存活率超过90%。本研究通过形貌匹配与分子识别的协同作用，成功构建了一种高效、低毒且抗干扰的CTC捕获平台，为癌症的早期诊断提供了潜在的技术途径。

## Abstract (en)

Circulating tumor cells （CTC） have emerged as crucial mediators in the metastatic cascade， offering invaluable insights as real-time liquid biomarkers for cancer progression， prognosis， and treatment response. Their exceptionally low concentration in peripheral blood， which typically ranges from a handful to a few dozen cells per milliliter amidst billions of background blood cells， poses formidable challenges for isolation and molecular characterization. Despite this， the efficient and specific capture of CTC holds tremendous potential for revolutionizing early cancer detection， dynamic monitoring of therapeutic efficacy， and guiding personalized treatment strategies. Currently， the primary technologies for CTC enrichment fall into two categories： immunoaffinity-based methods that employ antibodies targeting epithelial surface markers such as epithelial cell adhesion molecule （EpCAM）， and label-free approaches that leverage physical properties including cell size， deformability， and density， exemplified by membrane filtration and centrifugal techniques. However， these conventional methods are hampered by several inherent limitations， including high operational costs， dependence on highly variable surface antigen expression， insufficient capture specificity leading to low purity， and significant interference from heterogeneous blood components such as leukocytes and platelets. Consequently， there is an urgent and growing need to develop novel functional materials and platforms that offer enhanced selectivity， robust stability in physiological conditions， excellent biocompatibility， and improved clinical applicability for the effective isolation and analysis of CTC. In this study， we innovatively integrate cell imprinting technology with a rational amino acid-based affinity strategy to develop a tryptophan-histidine-arginine （WHR） tripeptide-functionalized cell-imprinted hydrogel for highly efficient and selective capture of CTC. The design leverages the unique properties of mesoporous silica nanoparticles （MSN） as carriers， which are first synthesized and then surface-modified with epoxy groups via silane coupling agents. The WHR tripeptide is subsequently grafted onto the MSN surface through a ring-opening reaction， yielding the WHR@SiO₂ composite material. This material demonstrates strong and specific binding affinity toward sialic acid （Neu5Ac） and sialylated glycopeptides （SGP）， which are overexpressed on the surface of many cancer cells. Building on this molecular recognition capability， a three-dimensional cell-imprinted hydrogel is fabricated using poly（ethylene glycol） dimethacrylate （PEGDMA） as the cross-linking backbone via free radical polymerization. The hydrogel is molded against SMMC-7721 template cells to create cavities that complement the target cells in size， shape， and surface topology， thereby enhancing capture efficiency through both physical and biochemical matching. Experimental results demonstrate that the WHR-modified hydrogel achieves a remarkable capture efficiency of up to 94% for SMMC-7721 cells， significantly outperforming hydrogels modified with individual amino acids such as tryptophan， histidine， or arginine alone. The system also exhibits excellent hemocompatibility， with minimal adsorption of human serum albumin （HSA）， below 5%， indicating superior anti-fouling properties in biological environments. In vitro cytotoxicity assessments confirm high biocompatibility， with cell viability exceeding 90% after 48 h of co-culture. Further characterization through scanning electron microscopy （SEM） and atomic force microscopy （AFM） reveals well-defined surface imprints that mirror the morphology of template cells， confirming the successful integration of topographical cues. The synergy between the physical structure of the imprinted cavities and the biochemical affinity of the WHR tripeptide is identified as the key factor contributing to the high capture performance， even at low cell concentrations （as few as 100 cells/mL）. In conclusion， this work presents a robust and efficient platform for CTC capture that combines cell imprinting for morphological recognition with WHR-mediated affinity for sialylated glycoproteins. The hydrogel demonstrates high selectivity， stability， and biocompatibility， offering a promising tool for clinical applications in liquid biopsy and early cancer detection. The modular design of the system also allows for adaptation to other cancer types by altering the peptide sequence or template cells， highlighting its broad potential in cancer research and diagnostics.

循环肿瘤细胞（circulating tumor cell， CTC）具有典型的肿瘤细胞表型特征，是肿瘤细胞获得侵袭能力的重要标志物^［1］^。基于CTC的诊断方法具有无创、快速、实时等显著优势^［2，3］^，相较于传统的组织活检、影像学和血清学检查更为便捷。更重要的是，完整的CTC能够提供比片段化的循环肿瘤DNA（circulating tumor DNA， ctDNA）和外泌体更全面的癌症发展信息^［4，5］^。然而，CTC检测面临巨大挑战^［6］^：在每毫升血液中，CTC仅有数个，却混杂在高达10^8^个红细胞（red blood cell， RBC）和10^5^个白细胞（white blood cell， WBC）中，这种极低的丰度使得传统细胞分析方法^［7，8］^难以有效识别。

目前，研究者主要通过两种策略富集CTC：一是基于生物物理特性的阴性富集^［9］^、抗黏附分子^［10］^和微过滤^［11］^等技术，这些方法虽能有效消除血液成分干扰，但往往导致CTC纯度、回收率和特异性降低^［1］^；二是基于免疫亲和的正向富集技术，通过特异性识别CTC表面标志物如：上皮细胞黏附分子（epithelial cell adhesion molecule， EpCAM）、vascular cell adhesion molecule 1（VCAM-1）或Glypican 3 （GPC 3）^［12，13］^，虽然富集效率较高，但存在成本昂贵、检出率不稳定、操作流程复杂等技术瓶颈^［14］^。因此，开发具有高特异性、良好稳定性且便于制备的CTC靶向分子，对推动CTC富集材料的发展具有重要意义。

蛋白质糖基化作为重要的翻译后修饰（post-translational modifications， PTM）类型^［15，16］^，在细胞增殖与分化过程中发挥着关键调控作用^［17-19］^。研究表明，肿瘤细胞表面聚糖的异常表达与癌症发生发展存在显著相关性^［20］^，这使得唾液酸化糖蛋白，尤其是唾液酸化聚糖（sialylated glycans， SG），成为CTC检测中极具前景的生物标志物。基于聚糖靶向受体的捕获技术为CTC检测提供了新思路，但SG的识别仍面临多重挑战^［21，22］^：首先，生物样本中SG丰度极低，且存在未修饰蛋白质和糖脂的严重干扰，导致其富集效率低^［23，24］^；其次，由于聚糖结构的高度复杂性和弱免疫原性特征，使得特异性抗体与人工受体的开发面临技术瓶颈^［25］^。这些因素共同制约着糖基化标志物在CTC检测中的应用。

生物样本的复杂性（如血液环境）对分子识别和特异性分子相互作用提出了严峻挑战，这在一定程度上制约着CTC的捕获效率。因此，材料设计中必须充分考虑目标细胞的形态特征和尺寸参数，以提升材料的特异性识别能力^［26，27］^。细胞印迹技术作为分子印迹技术的重要拓展^［28］^，在生物医学与材料科学交叉领域的发展历程中占据了重要地位^［29，30］^。早期分子印迹研究主要聚焦于小分子^［31］^、肽类^［32］^及蛋白质^［33］^，而细胞印迹的发展相对滞后^［34］^。随着细胞检测、分离及疾病诊断需求的日益增长，开发高效、精准的细胞识别材料已成为亟待解决的问题^［35，36］^。在此背景下，细胞印迹技术应运而生。该技术通过巧妙结合细胞形貌特征与分子识别机制，成功克服了传统抗体依赖技术存在的稳定性差、成本高、制备周期长等局限性，在生物医学应用中展现出广阔的应用前景^［37］^。然而，尽管细胞印迹材料具有诸多优势，但在实际开发过程中仍面临诸多技术挑战^［38］^。目前，现有细胞印迹材料普遍存在尺寸匹配精度不足^［39］^、生物相容性欠佳^［40］^等问题，这些因素严重制约了目标细胞的捕获效率和检测准确性。

为解决上述难题，我们创新性地设计并制备了一种色氨酸-组氨酸-精氨酸（tryptophan-histidine-arginine， Try-His-Arg， WHR）三肽修饰的水凝胶细胞印迹材料，用于实现CTC的高效捕获（图1）。首先制备多孔结构的介孔二氧化硅纳米颗粒（mesoporoussilicananoparticle， MSN），通过表面修饰成功接枝WHR三肽，验证其对唾液酸化糖肽（sialylatedglycopeptide， SGP）的特异性富集能力。实验结果表明，WHR三肽能够精确识别并区分唾液酸化与非唾液酸化聚糖。在存在500倍牛血清白蛋白的干扰条件下，WHR三肽仍可高效富集21条SGP，这一性能显著优于我们先前报道的组氨酸-组氨酸（histidine-histidine， HH）二肽（在10倍牛血清白蛋白干扰下富集16条SGP）^［20］^。此外，生物膜干涉实验显示，WHR三肽与靶标分子的解离常数（dissociation constant， *K*_d_）为26 μmol/L，明显低于HH二肽（0.8 mmol/L）^［20］^以及单一氨基酸色氨酸（tryptophan， Try， 9.6 mmol/L）、组氨酸（histidine， His， 3.5 mmol/L）、精氨酸（arginine， Arg， 3.8 mmol/L）的*K*_d_，充分证实了三肽修饰带来的唾液酸结合能力提升。基于这些发现，我们提出WHR三肽可能可以通过特异性识别CTC表面过度表达的唾液酸糖蛋白，从而实现高效细胞捕获的设想。

**图1:**
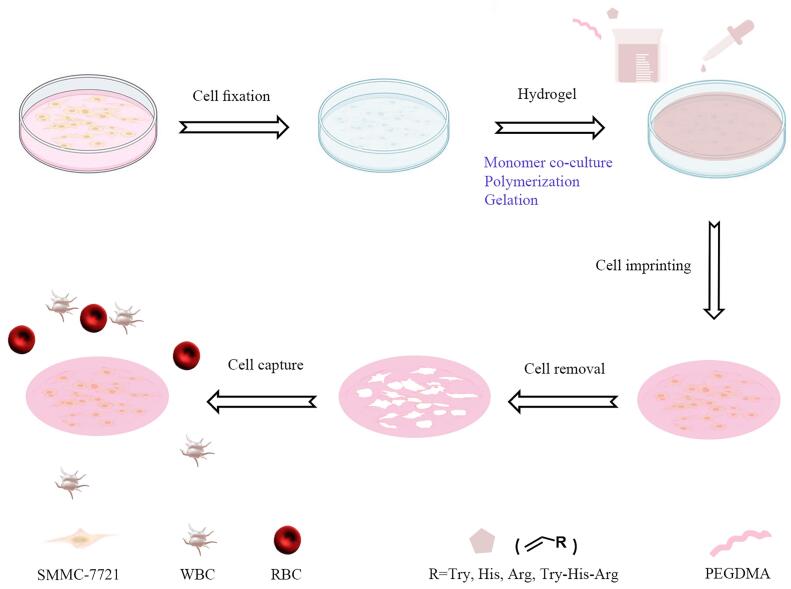
细胞印迹与氨基酸亲和协同水凝胶的制备及其对SMMC-7721细胞的捕获

为验证这一假设，我们进一步构建了WHR修饰的细胞印迹水凝胶材料，并对其生物相容性及CTC捕获性能进行评价。实验数据表明，该WHR水凝胶不仅展现出优异的生物相容性，而且对SMMC-7721细胞的捕获效率可达到94%。本研究设计的WHR三肽修饰细胞印迹水凝胶材料，为CTC检测提供了新的技术思路，有望推动癌症早期诊断领域发展更简便、高效的检测方法。

## 1 实验部分

### 1.1 仪器、试剂与材料

FreeZone 2.5L型冷冻干燥机（美国Labconco公司）；Dionex UltiMate 3000 RSLCnano系统的LTQ-Orbitrap Elite质谱仪（美国Thermo公司）；Nano ZS型纳米粒度仪（英国Malvern公司）；JEM-2100型透射电子显微镜（日本JEOL公司）；Avance型核磁共振波谱仪（400 MHz，德国Bruker公司）；UV-2550型紫外可见分光光度计（日本Shimadzu公司）；Multiskan GO型酶标仪（美国Thermo公司）。

十六烷基三甲基氯化铵（CTAC）、三乙醇胺（TEA）、正硅酸四乙酯（TEOS）、（3-缩水甘油丙氧基）三甲氧基硅烷（GPTMS）、噻唑蓝（MTT）、过硫酸钾（KPS）、碳酸氢铵（NH_4_HCO_3_）和尿素（纯度95%）购自上海麦克林生化科技有限公司。WHR（纯度95%）购自南京杰肽生物科技有限公司。甲基丙烯酰氯、乙腈（ACN）、甲酸、唾液酸（Neu5Ac）、*N*-乙基-*N*′*-*（3-二甲氨基丙基）碳二亚胺、*N*-羟基琥珀酰亚胺、Try、His和Arg（纯度95%）购自上海阿拉丁化学试剂有限公司。胎球蛋白（fetuin）、牛血清白蛋白（BSA）、人血清白蛋白（HSA）、二硫苏糖醇（DTT）、碘代乙酰胺、胰蛋白酶和聚乙二醇二甲基丙烯酸酯（PEGDMA）（纯度95%）购自美国Sigma-Aldrich。其他试剂购自上海国药集团化学试剂有限公司。此外实验用水为美国Millipore公司Milli-Q超纯水系统制备的超纯水。新鲜血液采集自新西兰兔，按照标准动物伦理操作流程从江南大学动物房获得（伦理号：JN.No0530b0480915 ［132］）。

C18硅球材料（Fresh Bioscience， SU2-096-025）和固相萃取小柱（Thermo Scientific， Eppendorf^TM^ GELoader^TM^ Tips， 10411193）用于糖肽富集实验，AR2G生物传感器（Sartorius， 18-5092）用于配体-受体亲和力验证。

### 1.2 色氨酸-组氨酸-精氨酸介孔二氧化硅球的制备

介孔二氧化硅球的制备：称取1 g CTAC与0.03 g TEA，置于三口烧瓶内，加入20 mL去离子水，于95 ℃下500 r/min加热1 h。随后将搅拌调整至1 500 r/min并稳定15 min，同时用1 mL注射器取0.8 mL TEOS。在1 500 r/min下将TEOS缓慢滴加进三口烧瓶中，滴加完毕后反应液继续95 ℃加热2.5 h。反应结束后关闭加热器，待反应液温度降至室温，将获得的二氧化硅微球以8 000 r/min离心15 min，并在超纯水和乙醇中洗涤3次分散和沉淀以去除残留的单体，并进行95 ℃真空干燥12 h获得CTAC@MSN。随后再将CTAC@MSN分散于100 mL含有5%浓盐酸的甲醇溶液中，于70 ℃回流24 h后8 000 r/min离心15 min，多次水洗得到MSN。

GPTMS@SiO_2_的合成：将500 mg MSN加入至5 mL含有GPTMS（0.5 mL）的无水甲苯溶液中。将反应混合物在90 ℃下搅拌24 h。将获得的二氧化硅微球（记为GPTMS@SiO_2_）以7 000 r/min离心5 min，并在甲苯和乙醇中洗涤3次分散和沉淀以去除残留的GPTMS，并进行冷冻干燥获得GPTMS@SiO_2_。

WHR@SiO_2_的合成：为了将Neu5Ac结合单元WHR固定在二氧化硅微球表面，将50 mg的GPTMS@SiO_2_、20 mg的WHR和15 μL的三乙胺加入至5 mL超纯水中。将混合物在65 ℃下搅拌24 h，得到WHR@SiO_2_。通过8 000 r/min离心10 min收集沉淀，并通过反复分散和沉淀循环依次用超纯水和乙醇清洗以去除残留化学物质，并进行冷冻干燥获得色氨酸-组氨酸-精氨酸介孔二氧化硅球（WHR@SiO_2_）。

### 1.3 色氨酸-组氨酸-精氨酸介孔二氧化硅球的表征

WHR@SiO_2_的形貌特征用电压为200 kV的TEM观察。具体操作如下：首先制备WHR@SiO_2_（1 mg/mL）水溶液，之后用10 μL移液枪将其滴在镀膜铜网上，待它们沉积数分钟后，用磷钨酸溶液负性染色，自然干燥后上机进行观察并拍照。WHR@SiO_2_（1 mg/mL）在水相中的粒径分布情况利用粒径仪在25 ℃条件下测定，每组实验重复3次，取平均值；另外在N_2_气氛下以10 ℃/min的加热速率进行热重分析（TGA）以证明WHR@SiO_2_成功合成。

### 1.4 蛋白酶解和除盐

将胎球蛋白（1 mg）和BSA（1 mg）分别溶解于尿素溶液（6 mol/L， 100 μL）中。以胎球蛋白为例介绍胰蛋白酶消化。DTT（5 μL， 200 mmol/L）溶解于尿素溶液中，在56 ℃下孵育45 min破坏胎球蛋白中的二硫键。然后加入碘乙酸（20 μL， 200 mmol/L），将溶液在室温下避光保存30 min。随后加入NH_4_HCO_3_溶液（50 mmol/L， 845 μL）和胰蛋白酶（30 μg），在37 ℃下反应16 h充分消化和裂解蛋白质。通过添加甲酸（10 μL）终止反应，并使用C18硅球材料填充固相萃取柱对样品进行脱盐。尿素、DTT和碘乙酸溶液的配制均用50 mmol/L的NH_4_HCO_3_溶液溶解。BSA采用相同的方法进行裂解。

称取1 mg的C18硅球材料，加入ACN（20 μL）混匀，均匀加压装填入塞有单层3M的C18膜片的固相萃取小柱。所得的SPE小柱首先用含0.1%甲酸的50%乙腈水溶液（20 μL）进行活化，再加入0.1%甲酸水溶液（20 μL）进行平衡，然后将上述酶解液加入至SPE小柱上。随后用0.1%甲酸水溶液（40 μL）冲洗各种盐类物质，0.1%甲酸的50% ACN水溶液（20 μL）释放待分析样品。最后冷冻干燥待分析样品并储存在-80 ℃冰箱中等待WHR@SiO_2_富集。

### 1.5 蛋白酶解和除盐

*N*-糖肽（*N*-linked glycopeptides， *N*-GP）富集采用固相萃取模式（SPE）进行，富集过程包括以下步骤：先将2 mg的WHR@SiO_2_悬浮于ACN溶液（20 μL）中。随后，用0.1%甲酸水溶液（20 μL）进行初步清洗，再用含0.5%甲酸的80% ACN水溶液（20 μL）进行平衡。之后，将胰蛋白酶肽（相当于10 μg的胎球蛋白和牛血清白蛋白的混合样品）在含0.5%甲酸的80% ACN水溶液（20 μL）中上样到WHR@SiO_2_材料上。再用含0.5%甲酸的75% ACN水溶液（20 μL）和含0.5%甲酸的70% ACN水溶液（20 μL）依次冲洗。最后，用5 mmol/L NH_4_HCO_3_的50% ACN水溶液（20 μL）洗脱吸附剂。收集最终的洗脱液进行LC-MS/MS分析。

### 1.6 糖肽富集

称取*N*-乙基-*N′*-（3-二甲氨基丙基）碳二亚胺（30 mg）和*N*-羟基琥珀酰亚胺（18 mg），溶解于400 μL超纯水中，转移至96孔板中，每孔加入200 μL溶液。将2个AR2G生物传感器分别在室温避光条件下置于含活化溶液的孔中，2 h后弃去孔中溶液，将1 mg的WHR溶解于1 mL超纯水中，加入孔中，将2个AR2G生物传感器分别置于含WHR溶液的孔中，在室温避光条件下静置反应24 h。利用WHR分子中的氨基基团，通过酰胺化反应将WHR共价接枝到羧基封端的AR2G表面。随后，用超纯水充分清洗AR2G表面，以去除未反应的样品。同时制备Neu5Ac溶液（3.2 mmol/L），并稀释得到不同浓度梯度（0.03、0.07、0.15、0.3、0.6、1和3 mmol/L）的测试溶液。将实验组（1个AR2G）分别暴露于不同浓度梯度的测试溶液中，在20 ℃条件下进行结合、解离和相互作用分析。同时，将对照组（另1个AR2G）暴露于超纯水中，在相同条件下进行对照实验。实验数据采用ForteBio Data Analysis 11.0软件进行分析。

### 1.7 核磁滴定

采用核磁共振氢谱（^1^H NMR）滴定实验研究WHR与Neu5Ac的分子间相互作用及结合位点。首先将WHR和Neu5Ac分别溶解于氘代二甲基亚砜（DMSO-d_6_）中，20 ℃条件下测定各自的^1^H NMR谱图。随后按照1∶1的等物质的量之比将WHR（16 mmol）缓慢加入Neu5Ac（16 mmol）溶液中，室温条件下混合均匀并静置12 h，之后20 ℃条件下测定其^1^H NMR谱图。通过对比分析WHR和Neu5Ac结合前后活泼氢的化学位移变化，确定二者相互作用的具体结合位点。

### 1.8 单体合成

将Try（2.04 g）、His（1.55 g）、WHR（100 mg）分别置于不同圆底烧瓶中，分别加入NaOH溶液（2.0、4.0、2.0 mmol/L），冰浴条件下搅拌30 min。随后使用注射器缓慢滴加甲基丙烯酰氯（1.94 mL、1.94 mL、240 μL），30 min后将反应体系移至室温继续搅拌24 h，以确保反应完全。反应结束后，以稀盐酸调节反应液pH至2.0，并加入饱和氯化钠溶液进行盐析。过滤除去不溶性固体后，以乙酸乙酯（15 mL）对水相进行3次萃取，合并有机相并经减压浓缩，所得粗产物用甲醇洗涤以去除沉淀。最终产物经浓缩并冷冻干燥，得到黄色粉末状甲基丙烯酰化色氨酸（2.6 g），产率为95.6%，相对分子质量为273.12；白色粉末状甲基丙烯酰化组氨酸（2.1 g），产率为93.2%，相对分子质量为224.10；对WHR冷冻干燥样品经过高效液相色谱进一步梯度纯化，收集馏分并冷冻干燥得到淡黄色粉末状甲基丙烯酰化色氨酸-组氨酸-精氨酸（84.0 mg），产率为74.3%，相对分子质量为566.29。

在圆底烧瓶中加入2.1 g Arg，加入20 mL饱和NaHCO_3_溶液，于冰浴下搅拌30 min。随后用1 mL注射器添加甲基丙烯酰氯（1.94 mL， 20 mmol），30 min后混合反应液室温环境持续搅拌24 h以确保反应完全。反应结束后，用稀盐酸将反应液pH值调节至1.0，并加入氯化钠饱和溶液进行盐析。过滤除去不溶性固体后，将滤液用乙酸乙酯洗涤3次，取水相用体积比为1∶1的乙酸乙酯-异丙醇混合溶液，萃取水相，旋蒸有机相，旋蒸得油状液体，加入适量异丙醇，后用无水MgSO_4_干燥该溶液，过滤除去MgSO_4_，水泵旋蒸至黏稠，再换用油泵旋蒸，最终得到白色粉末状甲基丙烯酰化精氨酸（1.9 g），产率为64.8%，相对分子质量为243.15。

### 1.9 水凝胶细胞毒性测试

将SMMC-7721细胞以5 000 个/孔的密度接种于96孔板中，于37 ℃、5% CO_2_的培养箱中孵育24 h，之后加入Try、His、Arg和WHR水凝胶（20 mg），每个浓度设置6个平行组，培养48 h。随后弃去培养基和样品，每孔加入100 μL的MTT（0.5 mg/mL）并孵育4 h。然后弃去MTT溶液，每孔加入100 μL二甲基亚砜溶解甲瓒晶体，将96孔板放置在酶标仪上，在570 nm处读取吸光度值（*A*）。各样品的细胞活力通过以下公式计算：细胞活力=*A*_sample_/*A*_control_×100%。其中*A*_sample_代表存在样品时的吸光度值，*A*_control_代表不存在样品时的吸光度值。

### 1.10 血液相容性测试

血液相容性是评估材料能否应用于生物医学实验的重要指标^［41］^。溶血率反映了材料与RBC接触时对细胞膜完整性的破坏程度，可通过测定溶液中释放的血红蛋白含量来评价材料的抗溶血性能。通常，溶血率越低，表明材料对RBC的损伤越小，血液相容性越高。具体操作如下：（1）先将所有器具用自来水冲洗后，于稀盐酸内浸泡24 h；以自来水冲洗干净后，依次使用蒸馏水和超纯水各浸泡30 min；最终经121 ℃高压蒸汽灭菌20 min后，取出备用。（2）在新西兰兔各项状态较好时严格按照江南大学动物伦理要求进行取血，在各组离心管中分别加入0.1 mL含柠檬酸钠（38 mg/mL）的新西兰兔新鲜全血（*V*_血液_/*V*_柠檬酸钠_=9∶1），再分别加入2 mL含有Try、His、Arg和WHR（100 mg）的磷酸盐缓冲溶液（PBS， pH 7.4），以只加入超纯水作为阳性对照，只加入PBS作为空白对照组。将样品于37 ℃水浴中孵育2.5 h后，在4 ℃条件下，8 000 r/min离心5 min，测定上清液在545 nm的吸光度。样品的溶血率按照公式进行计算：溶血率=［（*A*-*A*_0_）/（*A*_1_-*A*_0_）］×100%，其中*A、A*_0_和*A*_1_分别为实验组、空白对照组和阳性对照组的吸光度。

### 1.11 统计学分析

所有数据均显示为每个样本的平均值±标准差，所有图表中均以误差线表示。使用GraphPad Prism软件（版本9.5.1，graphpad-prism.com，美国）进行统计学分析。

## 2 结果与讨论

### 2.1 WHR@SiO_2_材料的合成

前期研究中，我们设计了一种基于HH配体的细胞印迹水凝胶，成功实现了对SGP的高效富集^［20］^。在该设计中，His因具有空间位阻小、氢键结合，且其p*K*_a_值与生理pH相近等优势^［42-44］^，能够在血液环境中动态识别Neu5Ac。然而，His与Neu5Ac的结合主要依赖氢键和静电相互作用，这可能导致二者亲和力不足。同时，His还可能与其他含有羧酸或羟基的分子结合，进而降低识别特异性。此外，当局部pH发生较大变化时，His的质子化状态也会随之改变，直接影响其与Neu5Ac的结合能力。

鉴于上述问题，我们重新设计了WHR用于Neu5Ac的识别。这一设计充分利用了His小巧灵活的氢键结合能力，为Neu5Ac结合提供了基础识别单元。同时，Try的芳香环结构，通过其*π*电子与Neu5Ac碳环骨架形成CH-*π*相互作用，增强了结合稳定性^［45］^。最后Arg的胍基所携带的正电荷，可与Neu5Ac的负电荷形成强效盐桥作用，不仅能够提高结合强度，还能提升识别特异性^［46，47］^。另外MSN具备规则孔道结构、高比表面积及2~200 nm可调孔径，碱性条件下制得的MSN孔径小、比表面积大，有利于复杂样品分离^［48，49］^。这里我们采用溶胶-凝胶法制备MSN，并进一步对其进行表面功能化修饰，以获取目标材料^［50］^（图2a）。具体制备流程如下：首先，以表面活性剂CTAC作为造孔模板，在三乙醇胺碱性水溶液中使四乙氧基硅烷发生水解反应，形成CTAC@MSN纳米粒子；随后，通过酸醇回流处理去除CTAC模板，得到具有介孔结构的MSN颗粒。接着，使用GPTMS对酸化处理后的MSN进行表面接枝。最后，利用GPTMS上的环氧基与WHR三肽分子中氨基的开环反应，成功将WHR三肽引入，制备得到具有Neu5Ac靶向潜力的WHR@SiO₂聚合物硅球。

**图2:**
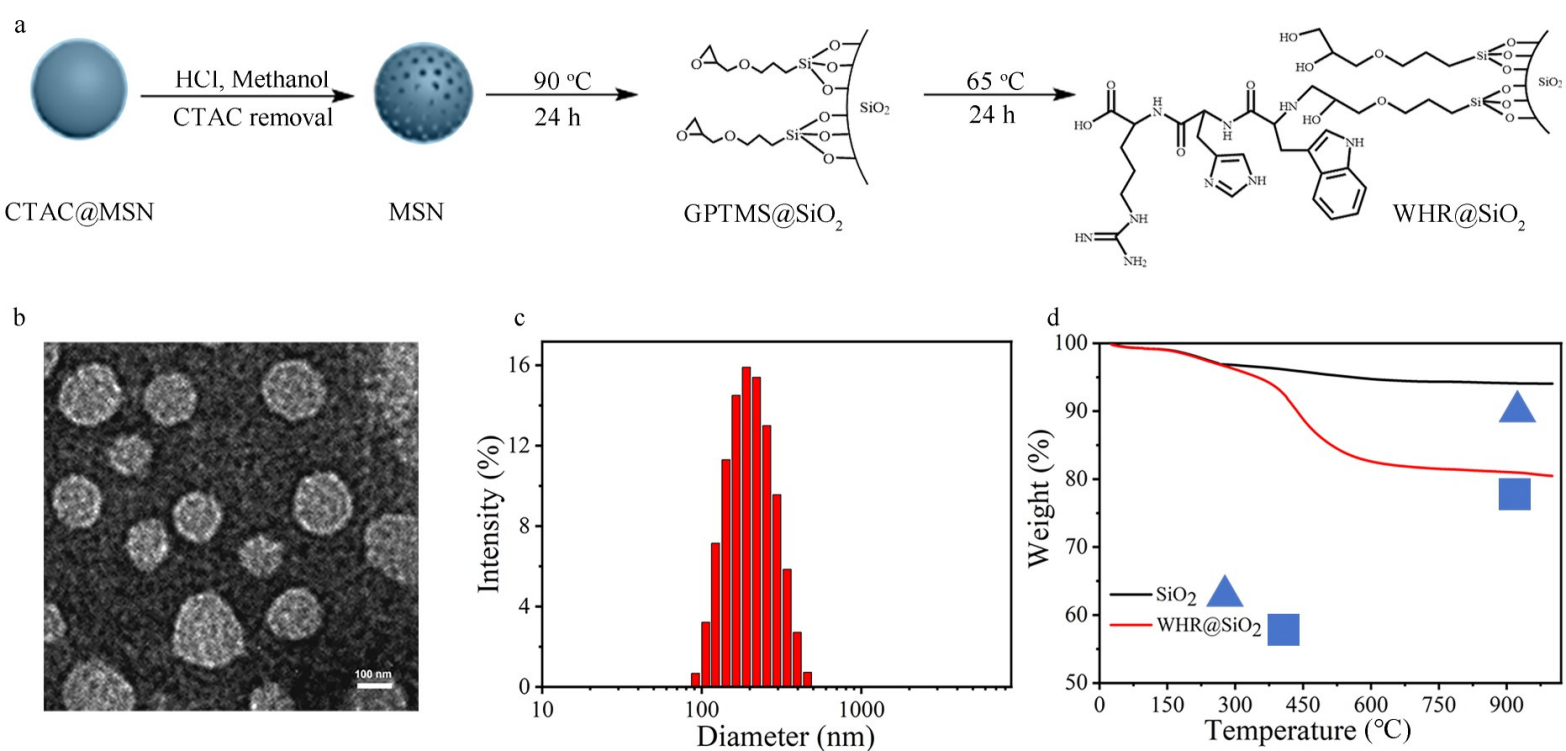
WHR@SiO_2_的表征

### 2.2 WHR@SiO_2_材料的表征

材料制备完成后，首先采用透射电子显微镜（TEM）对WHR@SiO₂的形貌和粒径进行表征。如图2b所示，WHR@SiO₂呈现均匀分布的球形结构，平均粒径约为150.0 nm，其表面可清晰观察到凹凸状的介孔孔道结构。值得注意的是，颗粒的外围存在约1 nm厚的白色光晕，这对应于材料表面修饰的有机壳层。为进一步验证粒径分布，我们通过动态光散射（DLS）对WHR@SiO₂进行了测试（图2c）。结果显示其水合粒径为190 nm，较TEM测试结果略大，这主要是源于水溶液中表面有机层的膨胀。随后进行的TGA（图2d）表明，当温度升至300 ℃时，MSN与WHR@SiO₂的重量损失基本一致，证实了WHR@SiO₂具有良好的热稳定性，这为其后续生物实验应用提供了保障。当温度继续升至900 ℃时，二者的残余质量分数分别为94.0%和80.4%，接枝率为13.6%。这一差异表明WHR@SiO₂表面有机组分含量明显增加，从而进一步验证了表面功能化改性的成功实施。

### 2.3 WHR@SiO_2_材料的糖肽富集

为考察WHR@SiO_2_材料在复杂体系中对富集*N*-GP的选择性^［51］^，我们以胎球蛋白与牛血清白蛋白酶解液的混合物为测试样品，测试WHR@SiO_2_材料在复杂背景下的*N*-GP富集性能。胰蛋白消化的混合样本产生了大量肽段，其中包括糖基化肽和未修饰肽。通过SPE富集流程，在上样、洗涤和洗脱过程中，有效去除了未糖基化肽的干扰。高分辨质谱分析显示，未经WHR@SiO₂材料富集的胎球蛋白/牛血清白蛋白（1∶500）胰蛋白消化混合物中，GP信号强度弱且检出数量有限（图3a）。而经WHR@SiO₂材料富集后，质谱图（图3b）中可清晰鉴定到21种唾液酸化*N*-GP（详细信息见表1），这些GP信号成为谱图中的主导峰，同时未修饰肽信号显著减弱，与图3a形成明显对比。该结果充分证明WHR@SiO₂材料对糖基化肽具有优异的选择性识别能力，展现了其出色的SGP富集性能。

**图3:**
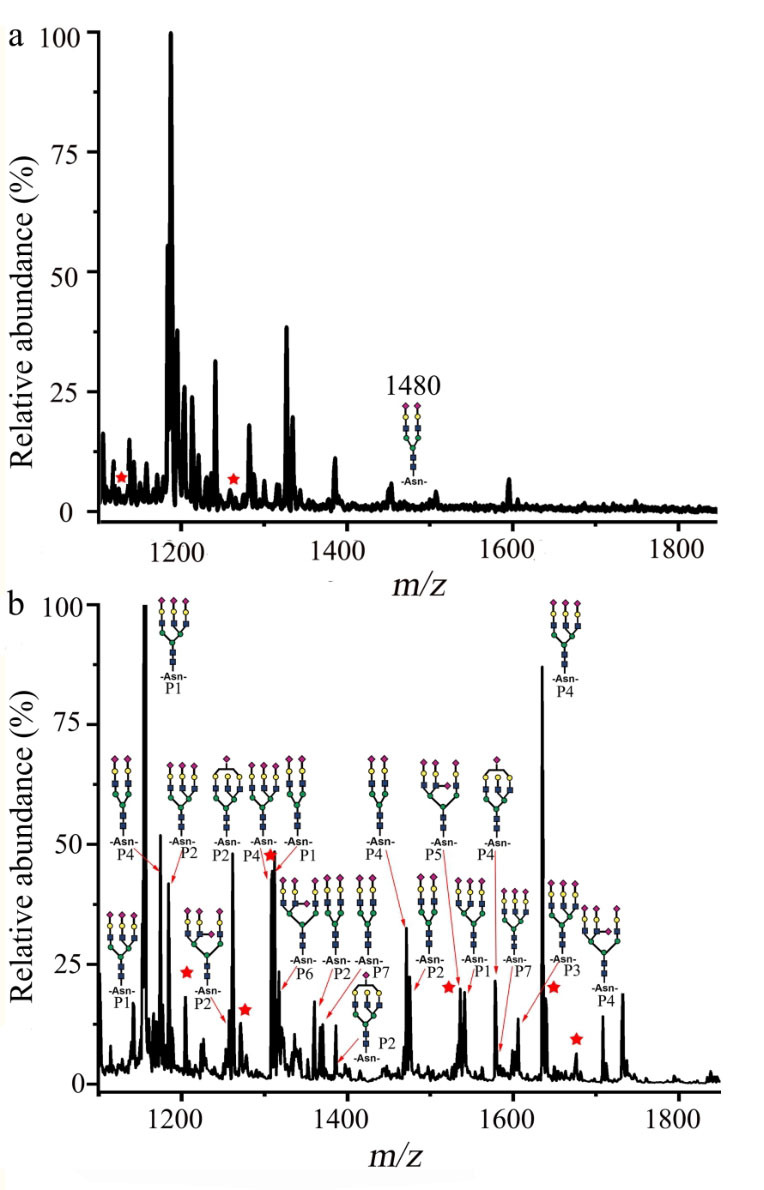
基于WHR@SiO_2_填充微SPE柱的*N*-GP富集策略

**表1:** WHR@SiO_2_材料的*N*-糖肽富集

Code name	Peptide sequence	Peptide *M*_r_	Glycan	*m/z* （charge state）
P1	LCPDCPLLAPLN（156）DSR（Cys_CM：146，149）	1740.8407	［Hex］5［HexNAc］4［NueAC］2	2224.1798， 1316.3402（3+）
			［Hex］6［HexNAc］5［NueAC］3	2880.8197， 1151.6651（4+）
			［Hex］6［HexNAc］5［NueAC］3	2879.0101， 1160.9563（4+）
			［Hex］6［HexNAc］5［NueAC］3	2879.0101， 1547.6060（3+）
P2	KLCPDCPLLAPLN（156）DSR	1868.9357	［Hex］5［HexNAc］4［NueAC］1	1932.9099， 1261.9485（3+）
			［Hex］5［HexNAc］4［NueAC］2	2224.1798， 1359.0385（3+）
			［Hex］6［HexNAc］5［NueAC］1	2298.2799， 1383.7385（3+）
			［Hex］6［HexNAc］5［NueAC］2	2589.5498， 1480.8285（3+）
			［Hex］6［HexNAc］5［NueAC］3	2880.8197， 1183.6889（4+）
			［Hex］6［HexNAc］5［NueAC］4	3172.0896， 1256.5063（4+）
P3	RPTGEVYDIEIDTLETTCHVLDPTPLAN（99）CSVR	3557.7250	［Hex］6［HexNAc］5［NueAc］3	2880.8197， 1605.8862（4+）
P4	RPTGEVYDIEIDTLETTCHVLDPTPLAN（99）CSVR（Cys_CM：89，100）	3671.7679	［Hex］5［HexNAc］4［NueAC］2	2224.1798， 1470.2369（4+）， 1176.3895（5+）
			［Hex］6［HexNAc］5［NueAC］2	2589.5498， 1561.5794（4+）
			［Hex］6［HexNAc］5［NueAC］3	2880.8197， 1634.3969（4+）， 1307.7175（5+）
			［Hex］6［HexNAc］5［NueAC］4	3172.0896， 1707.2144（4+）
P5	VVHAVEVALATFNAESN（176）GSYLQLVEISR	3016.5738	［Hex］6［HexNAc］5［NueAC］4	3172.0896， 1543.4159（4+）
P6	VWPRRPTGEVYDIEIDTLETTCHVLDPTPLANCSVR	4096.0266	［Hex］5［HexNAc］4［NueAC］3	2513.878， 1319.3861（5+）
P7	KLCPDCPLLAPLNDSR（Cys_CAM：146，149）	1868.9357	［Hex］5［HexNAc］4［NueAC］2	2222.7826， 1366.2372（3+）
			［Hex］6［HexNAc］5［NueAC］3	2879.0101， 1577.6524（3+）

P： glycopeptide； Hex： hexoses； HexNAc： hexosamines； NueAC： sialic acid.

### 2.4 WHR对唾液酸的亲和力评估

为了进一步验证WHR与Neu5Ac的结合亲和力，我们采用生物膜干涉技术（biolayer interferometry， BLI）和^1^H NMR滴定实验进行了研究。BLI技术凭借其高通量、高灵敏度等优势，是方便、快捷研究小分子-靶标相互作用的工具。与传统方法相比，BLI技术能够有效规避小分子快速解离导致的假阴性结果，同时避免标记物诱导的靶分子构象变化所产生的假阳性问题，从而准确地测定*K*_d_^［52］^。

实验结果显示，在动态响应曲线（图4a）中，Neu5Ac在WHR修饰的传感器表面表现出快速而强烈的吸附行为，对应较高的结合速率常数（*K*_on_）；同时，在WHR洗脱过程中观察到较低的解离速率常数（*K*_off_）。非线性拟合数据（图4b）计算得到WHR与Neu5Ac相互作用的*K*_d_值（*K*_off_/*K*_on_）为26 μmol/L。相比之下，Try、His和Arg单体与Neu5Ac相互作用的*K*_d_值分别为9.6、3.8和3.5 mmol/L，均显著高于WHR。这些实验数据有力证明了WHR与Neu5Ac之间具有特异的强结合亲和力。

**图4:**
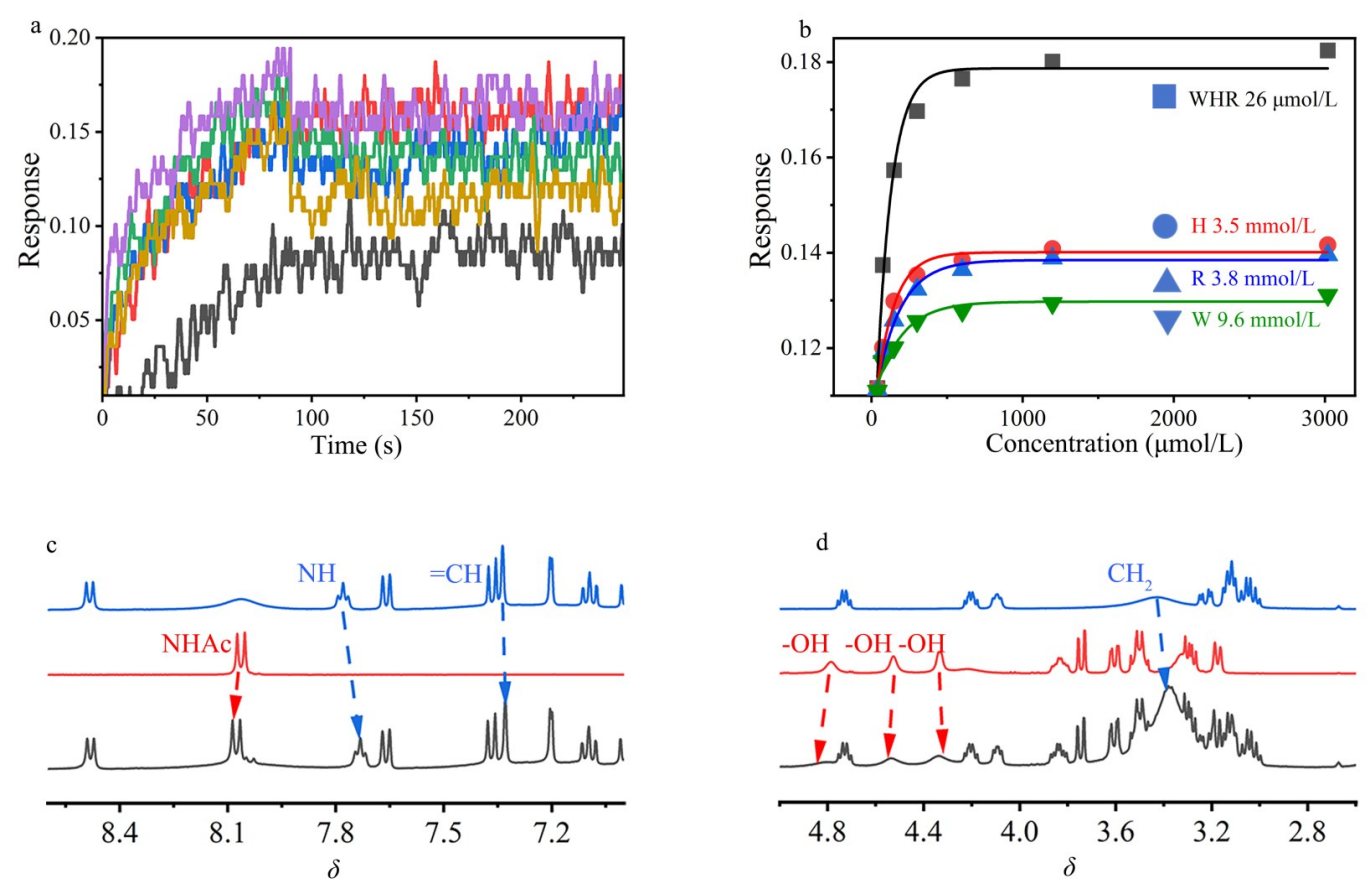
WHR和Neu5Ac的亲和力测定

为进一步验证这一结论，我们进行了^1^H NMR滴定实验（图4c~d）。谱图分析显示：WHR的氨基经取代后电负性减弱，化学位移向高场移动，同时C-H在7.35和3.4处的信号偏移并增强；Neu5Ac骨架中NHAc质子由于吸电子效应，在8.1处呈现明显的低场位移，而C-H质子在4.8~4.2范围内的羟基峰不仅消失还表现出低场移动特征。这些化学位移变化为WHR与Neu5Ac的分子间相互作用提供了实验证据。

### 2.5 水凝胶的制备和生物性能评价

基于WHR对Neu5Ac及SGP表现出的高亲和结合特性，并考虑到肿瘤细胞表面普遍存在异常高表达的SG^［53］^，我们选用生物相容性单体PEGDMA作为水凝胶骨架。通过对Try、His、Arg 3种氨基酸及其三肽WHR进行烯基化修饰，成功制备了4种氨基酸功能化水凝胶（Tryg、Hisg、Argg和WHRg）。如图5a所示，即使在PBS中浸泡48 h后，所得水凝胶仍能保持表面平整，未出现明显溶胀现象，表明该材料具有优异的形态稳定性，适用于生理环境下的生物医学应用。

**图5:**
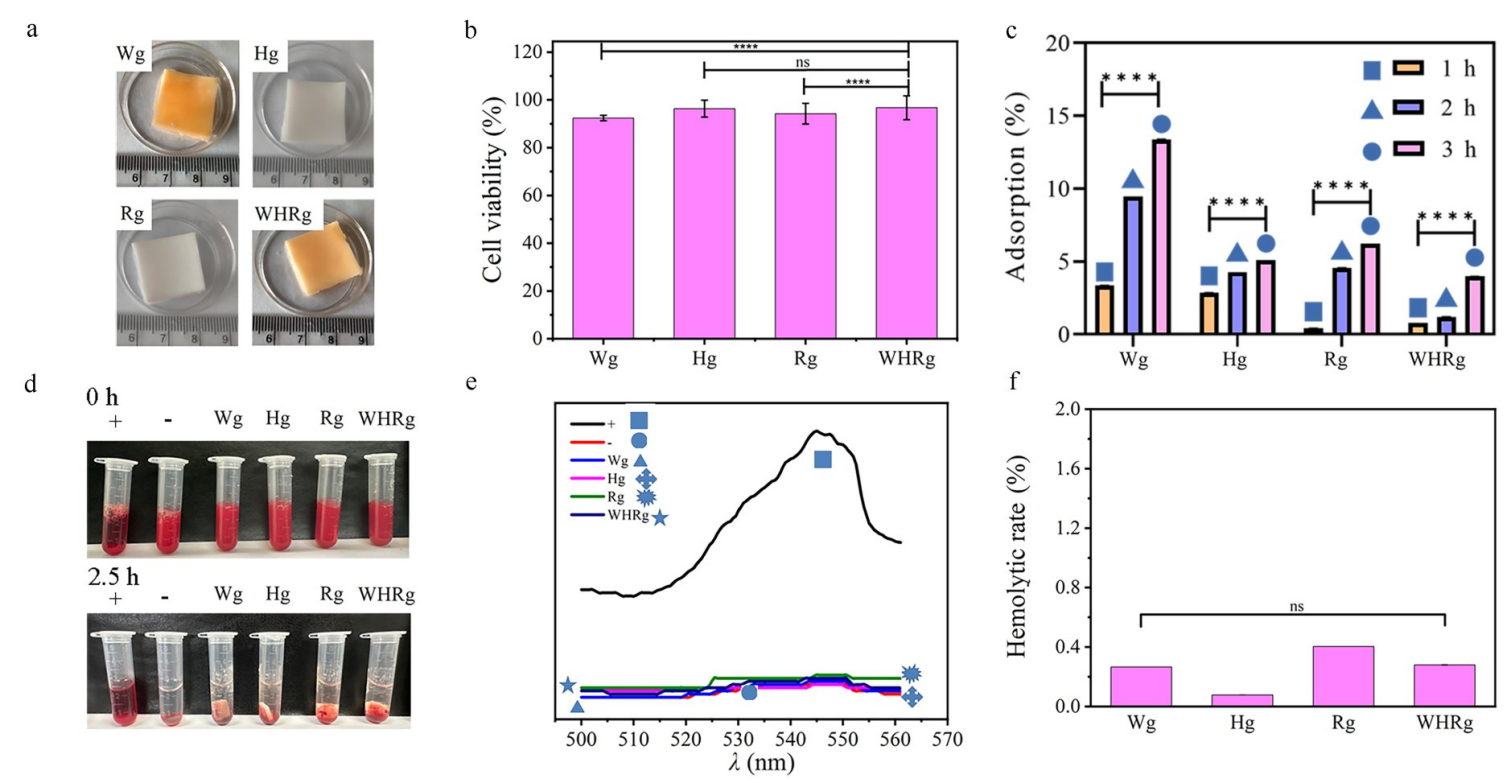
Wg、Hg、Rg、WHRg水凝胶的生物学评价

为了评估水凝胶的生物相容性，我们检测了它们对SMMC-7721细胞的影响（图5b）。体外培养实验显示，经过48 h共培养后，Tryg、Hisg、Argg和WHRg修饰水凝胶组的细胞存活率均维持在90%以上。这一结果证实，氨基酸修饰并未影响PEGDMA水凝胶基质固有的生物安全性，功能化改造后的材料仍保持优良的细胞相容性，符合生物医用材料的基本要求。

考虑到水凝胶在血液环境中的应用需求，我们重点考察了其对血浆主要成分HSA的吸附特性。HSA作为血浆中最丰富的蛋白质（质量浓度35~50 g/L，占血浆总蛋白的55%~60%），其吸附行为能有效反映材料的抗污能力^［54，55］^。通过动态监测水凝胶与HSA共孵育体系中的蛋白浓度变化，我们计算了各组材料的蛋白吸附能力。如图5c所示，WHRg修饰水凝胶在3 h孵育后的蛋白吸附率低于5%，显著优于单一氨基酸修饰组，表明WHRg具有优异的抗蛋白污染能力。

最后，我们通过溶血实验评价了4种水凝胶对RBC的影响。实验结果显示（图5d），在2.5 h水浴孵育后，阳性对照组（超纯水）出现明显溶血，溶液呈红色；而各实验组溶液经离心后，RBC均沉积于管底，未见明显溶血现象。紫外全波段扫描（图5e）在545 nm处检测了各组血红蛋白的特征吸收峰。定量分析显示（图5f），所有实验组的溶血率均低于0.5%，满足生物医用材料对血液相容性的要求。

扫描电子显微镜（scanning electron microscope， SEM）形态学观察（图6a和6b）显示了阳性组与阴性组RBC稀释溶液在硅板上的状态，其中阳性组由于RBC破裂，血红蛋白流出，因此未看到任何形貌，而阴性组由于在PBS中维持了渗透压的平衡，RBC未涨破，SEM图像清楚地看到了独属于RBC的凹形细胞形貌。图6c~6f显示了Tryg、Hisg、Argg和WHRg 4种水凝胶材料在血液稀释溶液中浸泡2.5 h后，经2.5%戊二醛固定处理的表面形貌。实验观察到所有水凝胶表面黏附的RBC数量均较少，这归因于材料优异的抗黏附特性。通过局部放大观察可见，附着在水凝胶表面的RBC保持着与阴性组相似的完整形态，表明这些材料与细胞具有良好的生物相容性。上述结果证明，我们制备的水凝胶材料不仅具有低细胞黏附特性，还展现出良好的生物安全性，为设计新型生物医用材料提供了理想平台。

**图6:**
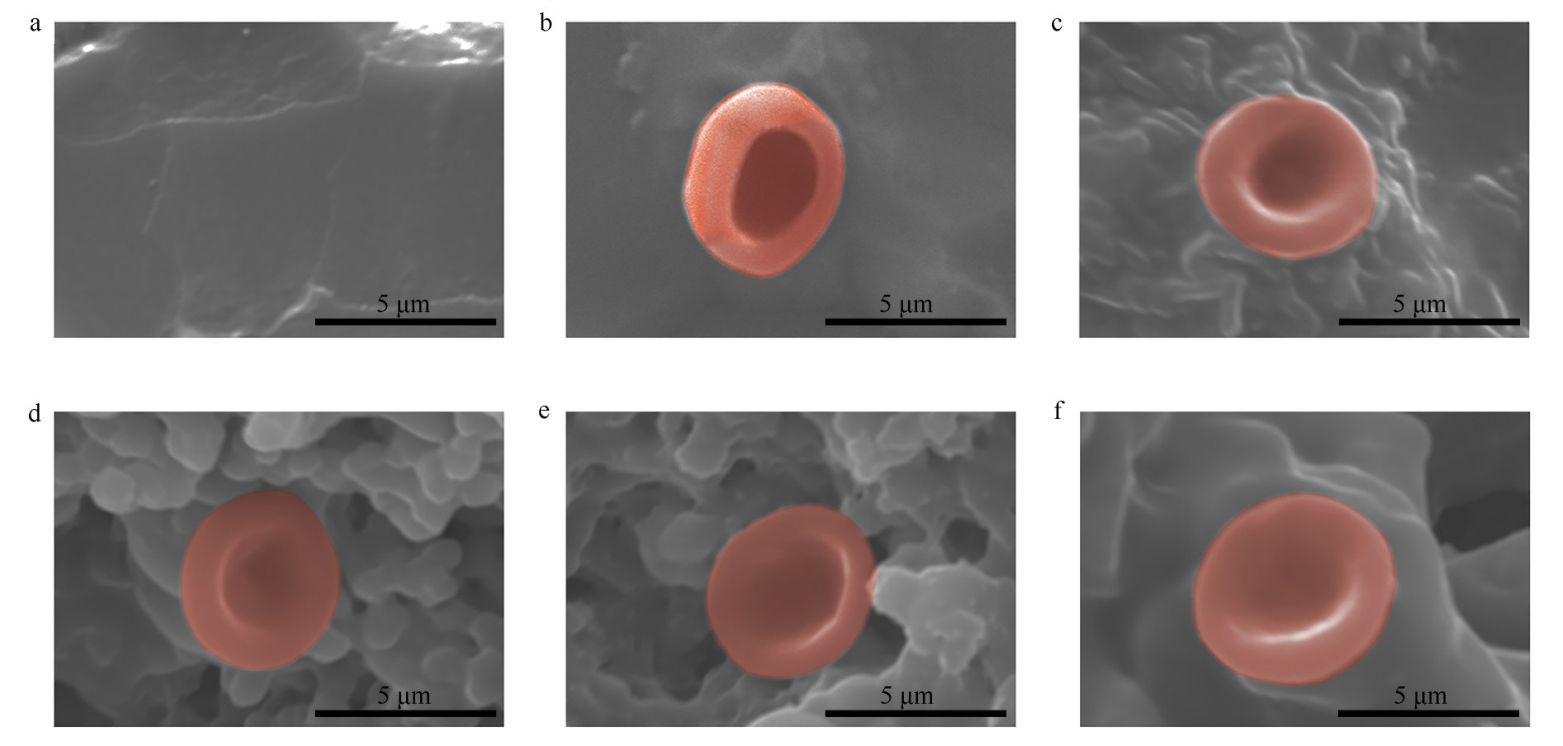
Wg、Hg、Rg、WHRg水凝胶对RBC的影响

### 2.6 细胞印迹水凝胶的形貌表征和性能测定

在完成生物安全性验证后，我们以SMMC-7721细胞为模板制备三肽聚合物基细胞印迹水凝胶。具体制备过程如下：首先使用2.5%戊二醛溶液对培养皿中的SMMC-7721细胞进行固定处理，以降低细胞膜流动性和分泌活性^［56］^。随后将功能单体与自由基引发剂KPS溶解于超纯水中，确保单体与细胞表面充分接触结合。经过2 h孵育后，将培养皿转移至烘箱中进行自由基聚合反应。聚合完成后，将所得水凝胶剥离并保持印迹面朝上，依次使用胰蛋白酶、超纯水和PBS进行清洗，以彻底去除残留的细胞模板、未反应单体及KPS（图1），最终制备得到4种细胞印迹水凝胶（Tryg+、Hisg+、Argg+和WHRg+）。

SEM（图7a）和原子力显微镜形貌学图片（图7b）显示，WHRg+水凝胶在去除模板细胞（10^6^）后，表面形成了平均尺寸为21 μm（长）×16 μm（宽）×1.5 μm（深）的椭圆形孔洞结构。当模板细胞数量降低至10^3^时，WHRg+水凝胶表面的印迹孔洞数量显著减少，表面粗糙度明显降低（图7c和7d）。并且模板细胞数量的显著降低导致细胞印迹水凝胶材料对CTC的捕获率从95%±0.3%降低至24%±1.8%。

**图7:**
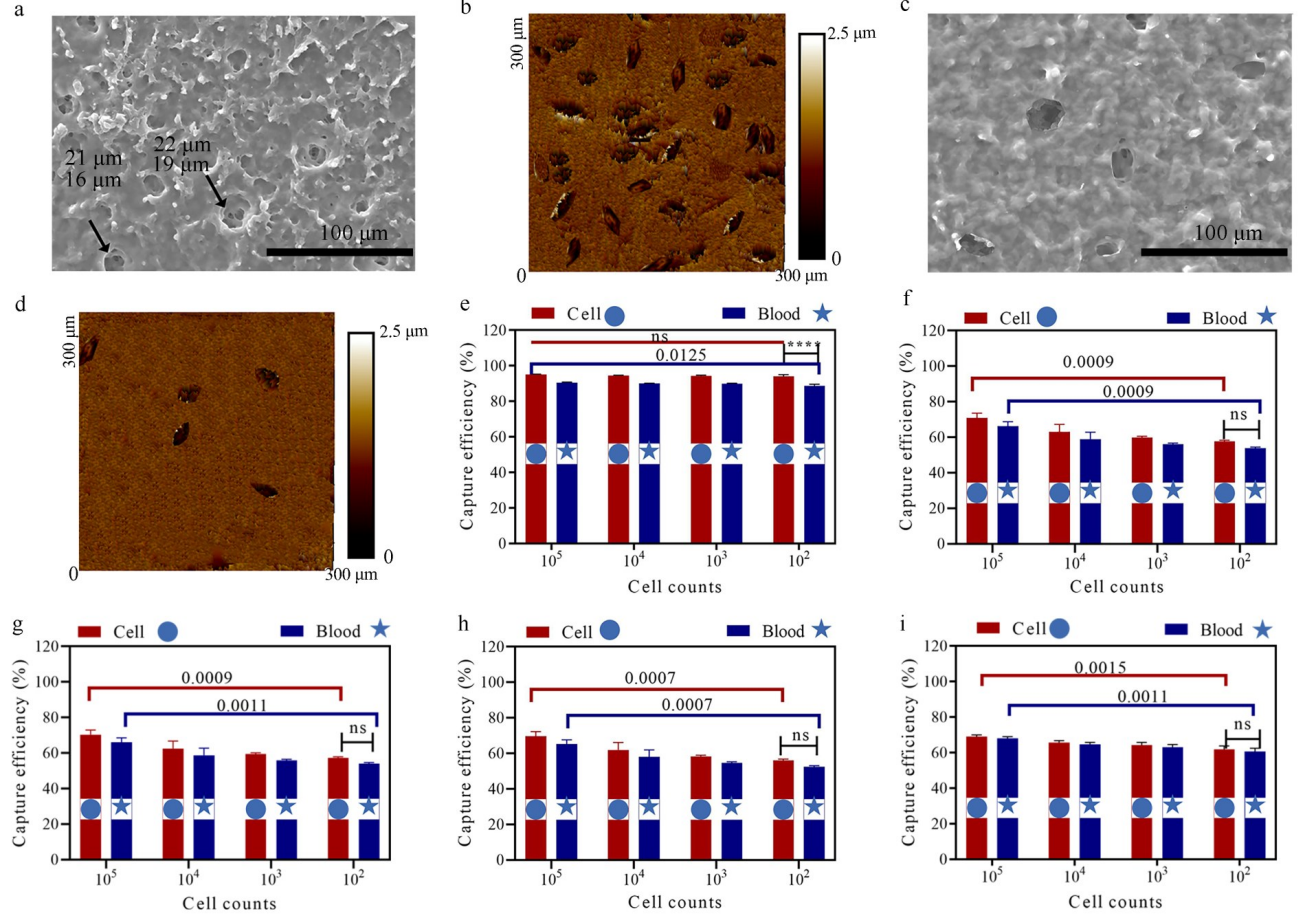
WHRg+水凝胶的表征和捕获性能评估

为了评估Tryg+（图7f）、Hisg+（图7g）、Argg+（图7h）和WHRg+（图7e）4种水凝胶的性能差异，我们分别设计了细胞水平和血液水平的细胞捕获实验。结果表明（图7e~h），4种水凝胶在全血中对SMMC-7721细胞的捕获效率与其在单纯细胞培养体系中的表现相当，这表明血液中的复杂成分不会影响水凝胶对目标细胞的富集能力。

值得注意的是，与单氨基酸修饰的水凝胶（Tryg+、Hisg+、Argg+）相比，WHRg+水凝胶展现出更优异的捕获性能：即使当细胞数量从10^5^降至10^2^时，其捕获效率仍保持在94%±0.9%（图7e）。相比之下，缺乏细胞形貌的WHRg水凝胶的捕获效率显著下降，10^2^细胞的捕获效率仅为62.0±1.9%（图7i），这一结果充分说明了形貌匹配对于细胞印迹材料实现高效捕获的关键作用。

## 3 结论

本研究成功开发了一种基于WHR三肽聚合物的细胞印迹水凝胶材料，用于高效捕获CTC。该材料通过协同整合细胞形貌匹配与多肽分子识别机制，实现了对目标细胞的高选择性捕获，并展现出优异的生物相容性、低蛋白吸附特性和良好的血液相容性。研究表明，该策略有效克服了传统CTC富集技术中抗体依赖性高、血液基质干扰强等技术瓶颈，为癌症的早期诊断提供了一种新型、高效且抗干扰的检测平台，具备良好的临床应用前景。
